# Editorial: The role of the microbiome in plant and soil health in a changing climate

**DOI:** 10.3389/fpls.2024.1491438

**Published:** 2024-10-02

**Authors:** Amita Kaundal, Anoop Kumar Srivastava, Dinesh Yadav

**Affiliations:** ^1^ Plants, Soils, and Climate, College of Agriculture and Applied Sciences, Utah State University, Logan, UT, United States; ^2^ Indian Council of Agriculture Research (ICAR)-Central Citrus Research Institute, Nagpur, Maharashtra, India; ^3^ Department of Biotechnology, Deen Dayal Upadhyaya Gorakhpur University, Gorakhpur, Uttar Pradesh, India

**Keywords:** microbiome, plant growth-promoting microbes, plant health, climate change, soil salinity, drought, heavy metal, elevated CO2

Industrialization during the mid-twentieth century drastically increased the earth’s temperature over the past few decades due to increased concentration of greenhouse gases, primarily carbon dioxide, by burning fossil fuels (Houghton, 2001). This rise in global temperature has led to extreme weather events worldwide, such as intense summers or harsh winters, and altered precipitation patterns, leading to prolonged droughts or severe floods (Ripple et al., 2022). The resulting environmental stress, a consequence of climate change, affects all living beings, including humans and plants. These stresses, especially the extreme heat and water conditions, negatively affect crop production and threaten food security (Ahmad et al., 2023). Soil salinity is another cause of concern due to elevated sea levels and extreme droughts (Munns and Tester, 2008; Sandhu and Kaundal, 2018). In nature, plants often face various stresses sequentially or simultaneously (Zandalinas and Mittler, 2022). Several studies reported the negative effect of combined environmental biotic and abiotic stresses on crop production and yield (Mahalingam, 2015; Ramegowda and Senthil-Kumar, 2015). The constant increase in world population, which is expected to reach 9 billion by 2050, demands an increase in food production by 70-85% (FAO, 2009). On top of that, anthropogenic activities and overuse of chemical fertilizers deteriorate soil health (Pahalvi et al., 2021; Santorufo et al., 2021). The root microbiome is one of the most diverse communities on the earth. It is mainly composed of rhizosphere microbes colonizing the immediate soil surrounding the plant root and endosphere microbes colonizing the internal tissues of the roots (Pascale et al., 2020; Bai et al., 2022) These microbes in the rhizosphere exhibit various plant growth-promoting activities such as nitrogen fixation, phosphate solubilization, siderophore, catalase, and IAA production and help plants’ growth and development (Mohanty et al., 2021; Ganesh et al., 2024). The plant growth-promoting bacteria significantly mitigates environmental abiotic and biotic stresses (Beneduzi et al., 2012; Kumar et al., 2019; Burlakoti et al., 2024).

The Research Topic, which has received diverse contributions, is a testament to the collaborative nature of scientific research. It is a collective effort to understand the role of the microbiome in plants and soil health in a changing climate, highlighting the role of soil microbes in mitigating salinity stress, drought, heavy metal toxicity, flooding, and elevated CO_2._ The first article suggested the potential of the cell-free supernatant in the novel *Devosia* sp. SL43 strain to sustain the soybean seed germination rate under salt stress (Monjezi et al.). Another study on soybean under elevated CO2 and flooding revealed higher bacterial and fungal diversity upon combined treatments compared to non-flooding control. The individual treatment of elevated CO2 and flooding revealed a significant abundance of *Chitinophaga, Clostridium*, and *Bacillus*. However, the combined treatments showed a considerable abundance of *Trichoderma* and *Gibberella*, offering hope for the future of plant and soil health in a changing climate (Coffman et al.). Another study focused is on phyllosphere epiphytic microbes’ diversity of five medicinal plants in summer and winter. The phyllosphere microbiome plays a significant role in plant physiological metabolism. The study revealed the seasonal effect on the bacterial and fungal phyllosphere compared to host species. The summer phyllosphere is more heterogeneous for microbial diversity than winter. The network connections of the bacterial and fungal communities significantly increased during season transition compared to plant connections. This study shed light on the understanding of the plant microbial community’s composition in small-scale agriculture and their ecological roles (He et al.). The article on the utilization of *Bacillus amyloliquefaciens* QST713-based product on potatoes revealed that this PGPB enhanced the potato yield and improved potato peel nutrient profile with a minor impact on the soil microbiome diversity (Adamo et al.). Another study on the PGPB revealed the biofertilizer and biocontrol properties of *Stenotrophomonas maltophilia* BCM. This PGPB significantly increased the wheat seed germination rate in the presence of two phytopathogens, *Rhizoctonia solani* and *Fusarium oxysporum*, as well as saline conditions. Genomic analysis of *S. maltophilia* revealed the presence of genes known for nutrient assimilation plant growth promoting traits such as plant growth and antifungal activities (Sharma et al.). The report on the impact of two PGPBs, *B. subtilis*, and *B. aryabhattai*, on mitigating salt stress in rice revealed the potential of these isolates for sustainable agriculture in the era of climate change. PGPB treatment in rice during salt stress improved the ionic and water balance, antioxidation defense, photosynthesis, nutrient uptake, and phytohormone production (Siddika et al.).

Drought and salinity, often in tandem, are an important climate conundrum affecting crop growth and development due to ominous auxin imbalance as a function of microbial diversity. However, functional microbial diversity is more impactful than mere numerical diversity, the former undergoing lesser reduction in water scarcity under organic production practices than conventional practices with assured irrigation (del-Canto et al.). The study on *Phaseolus vulgaris* recommends organic management rather than using agrochemicals to maintain enhanced rhizobia abundance, nodulation, and diversity (del-Canto et al.). Efforts must be made to develop sustainable and eco-friendly approaches for preserving and strengthening soil microbiota biodiversity.

Further, it has been recommended that many microbes as auxin-producing endophytes are reported to neutralize drought and salinity through auxin balance with coordinated auxin biosynthesis involving plant-indigenous auxin, microbes-associated auxins, and carriers of auxin transporters, apart from upregulation of stress-induced auxin-responsive microbial genes (Mal and Panchal). The intervention of omics-driven research in understanding the action mechanism and interaction of plants and associated plant rhizobacteria has been nicely reviewed (Verma et al.). The revelation of omics-based adaptive regulatory mechanisms underlying the plant adaptation under microbes-mediated abiotic stress reduction with improved plant nutrition as the second line of plant defense has been reported (Verma et al.). In another review, the potential of plant growth-promoting microorganisms for salinity tolerance in plants has been elucidated (Acharya et al.). Interestingly, rhizosphere microbes, as the second genome, put forth stressing plant defense through the elevated supply of growth-promoting hormones such as auxins, gibberellins, and cytokinins, coupled with a reduced level of stress causing ethylene, thereby striking a balance osmoprotectant secretion and further oxidative cellular damage (Acharya et al.). An interesting review highlighting the microbial intervention in the remediation of heavy metal toxicity, emphasizing the mechanism involved, ensures better rhizosphere health resilience (Tang et al.). Further, attempts have been made to enlist diverse approaches, including the recent nanotechnology, to improve the microbial remediation of heavy metal-polluted soils.

While developing a combative strategy against drought and salinity, photobomb-induced soil legacy effects (developing functional bridge accommodating pathogenic microbes, antagonists, and repeated recruitment of fresh microbial diversity, all collectively surviving through competitive coexistence) featuring rhizosphere secretions, non-preferential salinity-tolerant microbes coupled with the use of halophytes are highly pivotal (Ma et al.).

In conclusion, this research topic has a significant collection of articles shedding light on the role of plants’ phyllosphere, rhizosphere, and endosphere microbiome in plant growth and development and soil health under critical environmental stresses.
